# What Impact Does Lack of Sleep Have on Mental Health?

**DOI:** 10.3390/brainsci16030284

**Published:** 2026-03-01

**Authors:** Haitham Jahrami, Khaled Trabelsi, Seithikurippu R. Pandi-Perumal

**Affiliations:** 1 Government Hospitals, Manama 329, Bahrain; 2 Department of Psychiatry, College of Medicine and Medical Sciences, Arabian Gulf University, Manama 329, Bahrain; 3 High Institute of Sport and Physical Education of Sfax, University of Sfax, Sfax 3000, Tunisia; trabelsikhaled@gmail.com; 4 Department of Movement Sciences and Sports Training, School of Sport Science, The University of Jordan, Amman 11942, Jordan; 5 Division of Research and Development, Lovely Professional University, Phagwara 144411, Punjab, India; pandiperumal2023@gmail.com; 6 Centre for Research and Development, Chandigarh University, Mohali 140413, Punjab, India

## 1. Introduction

Mental illnesses represent a significant public health challenge on a global scale, contributing substantially to morbidity, mortality, disability, and impaired health-related quality of life, while exacerbating regional health inequities [1].

A clinically prominent comorbidity is the high frequency of sleep disturbance across nearly all developmental and psychiatric illnesses, functioning as a fundamental pathophysiological mechanism that can predispose to or perpetuate neuropsychiatric conditions [2]. Data indicate that insomnia affects about 16% of the general population worldwide, creating a massive global burden [3]. Research established that the relationship between sleep and neuropsychiatric disorders is characterized by bidirectional causality [4], where sleep disruption is not merely a symptom but a transdiagnostic correlate that often establishes a causative role in the etiology of mental illness [5].

Current evidence highlights that sleep quality serves as a critical mediator linking various behavioral and biological traits to mental health outcomes [6,7]. For instance, while evening chronotypes are often associated with higher rates of depression and anxiety, research shows that this relationship is fully mediated by poor sleep quality; the biological clock’s timing is less critical than the resulting sleep disruption [8].

The long-term mental health consequences of chronic sleep deprivation are particularly evident in the context of neurodegenerative disorders [9]. Nearly 44% of Alzheimer’s disease (AD) patients suffer from significant chronic sleep impairments, and up to 90% of all dementia patients experience some form of sleep disruption [10].

## 2. An Overview of the Published Papers in the Special Issue

Our Special Issue presents a multidisciplinary exploration of how sleep disruption serves as a critical mediator for various psychological and neurobiological conditions. A primary focus within this collection is the re-evaluation of traditional risk factors in favor of sleep-centric models. Chauhan et al. (2024) examined the long-standing association between evening chronotypes and poor mental health among young adults in the UK and Germany. Their research demonstrates that the relationship between an individual’s natural preference for evening activity and symptoms of depression, anxiety, and stress is fully mediated by poor sleep quality. By illustrating that “eveningness” itself is not a direct cause of mental illness when sleep quality is maintained, this paper offers a significant clinical insight: therapeutic interventions should prioritize circadian alignment and sleep restoration rather than attempting to alter an individual’s natural biological clock.

The influence of the digital age on sleep perception is examined by Jahrami et al. (2024), who present the first population-level prevalence estimates for orthosomnia—an unhealthy preoccupation with achieving “perfect” sleep as dictated by wearable tracking devices. Utilizing a novel classification algorithm, the study identified a conservative prevalence rate of 3% within the general population, revealing that these individuals experience significantly higher insomnia severity and sleep-related anxiety. This research underscores a paradoxical behavioral loop in which technology designed to monitor health becomes a source of technology-induced sleep anxiety. Consequently, healthcare providers must begin screening for obsessive device usage as a potential contributor to persistent insomnia.

In examining long-term cognitive outcomes, Niazi et al. (2025) provide a comprehensive review of the bidirectional relationship between chronic sleep deprivation and Alzheimer’s disease (AD). The authors elucidate how insufficient sleep promotes neuroinflammation and the accumulation of amyloid-*β* plaques and tau tangles, while AD pathology concurrently disrupts sleep architecture. The paper’s notable contribution lies in its neurobiological synthesis and the recommendation of Omega-3 fatty acids as a neuroprotective intervention. By mitigating neuroinflammation and enhancing synaptic function, Omega-3 supplements are proposed as a promising strategy for addressing the comorbidity of sleep deprivation and neurodegenerative decline.

The intersection of sleep, behavior, and culture is examined by Miller et al. (2025), who investigated Hispanic emerging adults to assess how insomnia symptoms influence self-regulated eating behaviors. Their findings indicate that increased insomnia severity significantly predicts lower control over food intake, a relationship that is partially mediated by acculturative stress. This study makes a valuable contribution by identifying sleep as a modifiable risk factor for problematic eating patterns in underserved populations. It highlights the necessity for mental health interventions for these groups to consider culturally specific stressors, such as the pressure to adapt to a dominant culture, which can exacerbate the adverse effects of sleep deprivation on behavioral health.

Chin and Xie (2025) examine the behavioral mechanisms through which Fear of Missing Out (FOMO) contributes to depression in emerging adults. Their findings indicate that insomnia symptoms act as a behavioral pathway, partially elucidating how the social anxieties of the digital age manifest as clinical depressive symptoms. A significant discovery in this research is that the FOMO–insomnia relationship is more pronounced in men than in women, suggesting that men may be more vulnerable to sleep-disrupting social hyperarousal. This paper enhances the Special Issue by positioning insomnia as a modifiable factor through which contemporary social pressures influence the psychological well-being of young adults.

## 3. Conclusions

The findings presented in this Special Issue underscore the profound and multifaceted impact of lack of sleep on mental health. Sleep disturbance, particularly insufficient duration, poor quality, or disorders such as insomnia, emerges as a critical transdiagnostic factor that not only accompanies but actively contributes to the onset, exacerbation, and maintenance of various psychiatric conditions or psychiatric symptoms.

The field is advancing rapidly, with ongoing research expected to refine our understanding of neurobiological mechanisms, identify biomarkers, and develop personalized interventions. Future efforts should prioritize longitudinal designs, diverse populations, and integrated approaches that bridge the artificial divide between sleep and mental health care. Ultimately, recognizing and treating sleep problems as a core component of mental health promotion could substantially reduce the global burden of psychiatric disorders.

## Data Availability

Not applicable.

## References

[1] Fan Y., Fan A., Yang Z., Fan D. (2025). Global burden of mental disorders in 204 countries and territories, 1990-2021: Results from the global burden of disease study 2021. BMC Psychiatry.

[2] Hyndych A., Koval K., Dzeruzhynska N., Mader E.C. (2025). Sleep and psychiatric disorders: Bidirectional interactions and shared neurobiological mechanisms. PLoS Ment. Health.

[3] Benjafield A.V., Sert Kuniyoshi F.H., Malhotra A., Martin J.L., Morin C.M., Maurer L.F., Cistulli P.A., Pépin J.L., Wickwire E.M. (2025). Estimation of the global prevalence and burden of insomnia: A systematic literature review-based analysis. Sleep Med. Rev..

[4] Crinion S., Morris D.W., Lopez L.M. (2024). Neuropsychiatric disorders, chronotype and sleep: A narrative review of GWAS findings and the application of Mendelian randomization to investigate causal relationships. Genes. Brain Behav..

[5] Sun X., Liu B., Liu S., Wu D.J.H., Wang J., Qian Y., Ye D., Mao Y. (2022). Sleep disturbance and psychiatric disorders: A bidirectional Mendelian randomisation study. Epidemiol. Psychiatr. Sci..

[6] Scott A.J., Webb T.L., Martyn-St James M., Rowse G., Weich S. (2021). Improving sleep quality leads to better mental health: A meta-analysis of randomised controlled trials. Sleep Med. Rev..

[7] Li Z., Zhong T., Meng X. (2025). A meta-analysis study evaluating the effects of sleep quality on mental health among the adult population. BMC Public Health.

[8] Zou H., Zhou H., Yan R., Yao Z., Lu Q. (2022). Chronotype, circadian rhythm, and psychiatric disorders: Recent evidence and potential mechanisms. Front. Neurosci..

[9] Yuan X.-L., Wang C.-Y. (2025). Sleep deprivation-induced cognitive impairment: Unraveling the role of neuroinflammation. Exp. Neurol..

[10] Wong R., Lovier M.A. (2023). Sleep Disturbances and Dementia Risk in Older Adults: Findings From 10 Years of National U.S. Prospective Data. Am. J. Prev. Med..

